# Clinical standards for antimicrobial stewardship in TB care

**DOI:** 10.5588/ijtldopen.25.0522

**Published:** 2025-12-10

**Authors:** T.T. Brehm, O.W. Akkerman, G. Sotgiu, S. Tiberi, K.-C. Chang, K. Dheda, R. Duarte, D. Vambe, Z.F. Udwadia, D. Chesov, M. Mendelson, A.M. Iswari Saktiawati, J. van Ingen, F.O. Eyuboglu, T. Tängdén, L.N. Quang Vo, N. Riccardi, C. Moschos, J.S. Friedland, T. Lillebaek, S.J. Chandy, J.A. Caminero, G. Thwaites, S. Gandra, K. Thursky, I.A. George, O. Konstantynovska, R. Fatima, J.-J. Yim, N. Kwak, I.D. Olaru, S.H. Gillespie, Y. Kherabi, S.H. Perl, E. Grønningen, C. Rodrigues, S. Bjerrum, F. Bange, V. Cox, D.M. Cirillo, F. Saluzzo, G.L. Hara, D. Wagner, N. Ismail, D.J. Sloan, I. Eshun-Wilsonova, M. Zeng, C. Cantero, T. Vasankari, A. Mandalakas, A. Kay, T. Ness, M.M. Torrico, G. Günther, L. Kuksa, L. Guglielmetti, A.L. García-Basteiro, G.B. Marks, C. Pulcini, C. Lange

**Affiliations:** 1 Department of Clinical Infectious Diseases, Research Center Borstel, Leibniz Lung Center, Borstel, Germany;; 2 German Center for Infection Research (DZIF), Partner Site Hamburg-Lübeck-Borstel-Riems, Borstel, Germany;; 3 Division of Infectious Diseases, I. Department of Internal Medicine, University Medical Center Hamburg-Eppendorf, Hamburg, Germany;; 4 Department of Pulmonary Diseases and Tuberculosis, University of Groningen, University Medical Center Groningen, Groningen, the Netherlands;; 5 Tuberculosis Center Beatrixoord, University of Groningen, University Medical Center Groningen, Haren, the Netherlands;; 6 Clinical Epidemiology and Medical Statistics Unit, Department of Medicine, Surgery and Pharmacy, University of Sassari, Sassari, Italy;; 7 StopTB Italia ODV, Milan, Italy;; 8 Blizard Institute, Barts and The London School of Medicine and Dentistry, Queen Mary University of London, London, UK;; 9 Tuberculosis and Chest Service, Centre for Health Protection, Department of Health, Hong Kong, China;; 10 Centre for Lung Infection and Immunity, Division of Pulmonology, Department of Medicine and UCT Lung Institute and South African MRC/UCT Centre for the Study of Antimicrobial Resistance, University of Cape Town, Cape Town, South Africa;; 11 Faculty of Infectious and Tropical Diseases, Department of Immunology and Infection, London School of Hygiene and Tropical Medicine, London, UK;; 12 EPIUnit, Instituto de Saúde Pública da Universidade do Porto, Porto, Portugal;; 13 Laboratório Associado para a Investigação Integrativa e Translacional em Saúde Populacional (ITR), Porto, Portugal;; 14 Instituto de Ciencias Biomédicas Abel Salazar - ICBAS, Universidade do Porto, Porto, Portugal;; 15 Instituto Nacional de Saúde Doutor Ricardo Jorge - INSA-Porto, Porto, Portugal;; 16 The Global Tuberculosis Program, Department of Pediatrics, Baylor College of Medicine, Houston, TX, USA;; 17 Baylor Children’s Foundation-Eswatini, Mbabane, Eswatini;; 18 P.D. Hinduja National Hospital and Medical research Centre, Mumbai, India;; 19 Discipline of Pneumology and Allergology, Nicolae Testemitanu State University of Medicine and Pharmacy, Chisinau, Moldova;; 20 Division of Infectious Diseases and HIV Medicine, Department of Medicine, University of Cape Town, Cape Town, South Africa;; 21 Department of Internal Medicine, Faculty of Medicine, Public Health, and Nursing, Universitas Gadjah Mada, Yogyakarta, Indonesia;; 22 Center for Tropical Medicine, Faculty of Medicine, Public Health, and Nursing, Universitas Gadjah Mada, Yogyakarta, Indonesia;; 23 Department of Medical Microbiology, Radboud University Medical Center, Nijmegen, the Netherlands;; 24 FOE Respiratory Clinic, Ankara, Turkey;; 25 Department of Medical Sciences, Uppsala University, Uppsala, Sweden;; 26 Friends for International TB Relief, Ha Noi, Vietnam;; 27 Department of Global Public Health, Karolinska Institutet, Stockholm, Sweden;; 28 TB Reference Center and Laboratory, ASST Grande Ospedale Metropolitano Niguarda, Milan, Italy;; 29 ‘Sotiria’ Hospital for Chest Diseases, Anti-Tuberculosis Department and Drug Resistant TB Unit, Athens, Greece;; 30 Institute of Infection and Immunity, City St. George’s, University of London, London, UK;; 31 International Reference Laboratory of Mycobacteriology, Statens Serum Institut, Copenhagen, Denmark;; 32 Global Health Section, Department of Public Health, University of Copenhagen, Copenhagen, Denmark;; 33 Department of Pharmacology and Clinical Pharmacology, Christian Medical College, Vellore, India;; 34 Pneumology Department, Universitary Hospital of Gran Canaria ‘Dr. Negrín’, Las Palmas de Gran Canaria, Spain;; 35 Director of Scientific Activities, ALOSA TB ACADEMY, Las Palmas, Spain;; 36 Oxford University Clinical Research Unit, Ho Chi Minh City, Vietnam;; 37 Division of Infectious Disease, Washington University School of Medicine, St. Louis, MO, USA;; 38 Associate Hospital Epidemiologist, Barnes-Jewish Hospital, St. Louis, MO, USA;; 39 Director RMH Guidance Group, Melbourne, VIC, Australia;; 40 National Centre for Antimicrobial Stewardship, Department of Infectious Diseases, University of Melbourne, Parkville, VIC, Australia;; 41 Centre for Health Services Research in Cancer, Peter MacCallum Cancer Centre, Melbourne, VIC, Australia;; 42 Department of Infectious Diseases and Clinical Immunology, V. N. Karazin Kharkiv National University, Kharkiv, Ukraine;; 43 Regional Phthisiopulmonological Center, Kharkiv, Ukraine;; 44 Department of Infectious Diseases, Imperial College London, London, UK;; 45 LLC ‘FH Clinic’, Kharkiv, Ukraine;; 46 Technical Advisor Consultant TB Strategic Planning and Global Fund Applications UNOPS;; 47 Department of Internal Medicine, Seoul National University College of Medicine, Seoul, South Korea;; 48 Division of Pulmonary and Critical Medicine, Seoul National University Hospital, Seoul, South Korea;; 49 Clinical Research Department, London School of Hygiene and Tropical Medicine, London, UK;; 50 School of Medicine, Division of Infection and Global Health, University of St Andrews, St Andrews, UK;; 51 Infectious and Tropical Diseases Department, Bichat-Claude Bernard Hospital, Assistance Publique-Hôpitaux de Paris, Université Paris Cité, Paris, France;; 52 Université Paris Cité, Inserm, IAME, Paris, France;; 53 Tuberculosis Departement, Ministry of Health, Jerusalem, Israel;; 54 Pulmonary Institute, Shamir Medical Center, Zerifin, Israel;; 55 Tuberculosis Clinic Rehovot, Maccabi Health Services, Rehovot, Israel;; 56 Centre for International Health, Department of Global Public Health and Primary Care, Faculty of Medicine, University of Bergen, Bergen, Norway;; 57 Department Lab Medicine, Hinduja Hospital, Mumbai, India;; 58 Department of Infectious Diseases, Copenhagen University Hospital, Rigshospitalet, Denmark;; 59 Institute for Medical Microbiology and Hospital Epidemiology, Hannover Medical School, Hannover, Germany;; 60 Johnson & Johnson, Titusville, NJ, USA;; 61 IRCCS San Raffaele Scientific Institute, Milan, Italy;; 62 Unidad de Infectología, Hospital Carlos G Durand, Buenos Aires, Argentina;; 63 Division of Infectious Diseases, Department of Internal Medicine II, Freiburg University Medical Centre, Freiburg, Germany;; 64 South African Medical Research Council Centre for Tuberculosis Research, Division of Molecular Biology and Human Genetics, Faculty of Medicine and Health Sciences, Stellenbosch University, Cape Town, South Africa;; 65 Department of Pulmonology, Radboud University Medical Center, Nijmegen, the Netherlands;; 66 Johnson & Johnson, Cape Town, South Africa;; 67 Department of Infectious Diseases, Children’s Hospital of Fudan University, National Children’s Medical Center, Shanghai, China;; 68 Service de Pneumologie, Département de Médecine, Hôpitaux Universitaires de Genève, Genève, Switzerland;; 69 Faculté de Médecine, Université de Genève, Genève, Switzerland;; 70 Department of Pulmonary Diseases and Clinical Allergology, University of Turku, Turku, Finland;; 71 Finnish Lung Health Association (FILHA), Helsinki, Finland;; 72 Department of Biological Sciences, University of Alaska Anchorage, Anchorage, AK, USA;; 73 Clínica de Tuberculosis, Instituto Nacional de Enfermedades Respiratorias Ismael Cosío Villegas, Mexico City, Mexico;; 74 Department of Pulmonology, Allergology and Clinical Immunology, Inselspital Bern, Bern University Hospital, Bern, Switzerland;; 75 Department of Clinical Sciences, School of Medicine, University of Namibia, Windhoek, Namibia;; 76 Tuberculosis and Lung Disease Clinic, Riga East University Hospital, Riga, Latvia;; 77 Department of Infectious, Tropical Diseases and Microbiology, IRCCS Sacro Cuore Don Calabria Hospital, Verona, Italy;; 78 ISGlobal, Hospital Clínic, Universitat de Barcelona, Barcelona, Spain;; 79 Centro de Investigação em Saúde de Manhiça, Maputo, Mozambique;; 80 Centro de Investigación Biomédica en Red de Enfermedades Infecciosas, Barcelona, Spain;; 81 Woolcock Institute of Medical Research, Ba Dinh District, Vietnam;; 82 Burnet Institute, Melbourne, VIC, Australia;; 83 Université de Lorraine, Inserm, INSPIIRE, Nancy, France;; 84 CHRU-Nancy, Centre Régional en Antibiothérapie du Grand Est AntibioEst, Université de Lorraine, Nancy, France;; 85 Respiratory Medicine and International Health, University of Lübeck, Lübeck, Germany;; 86 Institute for Infection Research and Vaccine Development (IIRVD), University Medical Center Hamburg-Eppendorf, Hamburg, Germany.

**Keywords:** tuberculosis, antimicrobial resistance, drug resistance, rifampicin resistance

## Abstract

**BACKGROUND:**

While antimicrobial stewardship (AMS) is essential for combating antimicrobial resistance (AMR), TB-specific AMS strategies remain poorly defined.

**METHODS:**

An international panel of 62 experts participated in a Delphi process. Using a 5-point Likert scale (5 = strong agreement; 1 = strong disagreement), participants evaluated 10 draft clinical standards developed by a core coordination team. A standard was adopted if ≥90% of respondents rated it three or higher, according to a predefined consensus threshold.

**RESULTS:**

All 10 standards reached the consensus threshold and were adopted: Standard 1, integration of TB into national AMR action plans; Standard 2, implementation of TB surveillance systems; Standard 3, education of health care providers, individuals affected by TB, and the public; Standard 4, integration of TB into AMS activities; Standard 5, establishment of expert consultation services; Standard 6, targeted testing and preventive treatment for individuals at risk for TB; Standard 7, access to timely and comprehensive drug susceptibility testing; Standard 8, prioritisation of efficacy, safety, and resistance prevention in TB treatment regimens; Standard 9, clinical and microbiological monitoring of treatment response; and Standard 10, assessment of adherence, drug exposure, and resistance in treatment failure.

**CONCLUSION:**

These clinical standards aim to support clinicians, programme managers, and public health authorities in implementing effective, TB-specific AMS strategies.

Antimicrobial resistance (AMR) is among the most urgent global health challenges of the 21st century. In 2021, an estimated 1.1 million deaths were directly attributable to bacterial AMR worldwide, and projections suggest that between 2025 and 2050, AMR could cause an additional 39 million deaths.^1^ TB is also a major contributor to the global AMR burden: in 2023, approximately 400,000 individuals were estimated to have developed multidrug-resistant or rifampicin-resistant TB (MDR/RR-TB) resulting in 150,000 deaths. The World Health Organization (WHO) included rifampicin-resistant *Mycobacterium tuberculosis* in its 2024 Bacterial Priority Pathogens List as one of only four pathogen groups identified as requiring urgent research, development, and strategic action to address AMR.^2^ Addressing AMR requires a multifaceted approach, with antimicrobial stewardship (AMS) playing a central role. AMS refers to coordinated strategies that promote the responsible use of antimicrobials to optimise clinical outcomes while minimizing toxicity and the emergence of resistance.^3,4^

Although drug-resistant TB (DR-TB) significantly contributes to the global AMR burden, most AMS efforts to date have focused on other bacterial pathogens. TB-specific AMS approaches remain poorly defined.^5^ To address this gap, we developed expert-driven guidance informed by a structured Delphi process involving an international panel of TB and AMS experts to reach expert consensus.^6^ The IJTLD Clinical Standards for Lung Health are intended to complement existing WHO and other international guidelines by providing a clinical framework to support their implementation.^7^ While grounded in universal principles, their successful adoption may require adaptation to local legal, organisational, and resource contexts.

## AIM OF THE CLINICAL STANDARDS

This consensus-based document defines 10 clinical standards to guide AMS in TB. The standards aim to promote responsible use of anti-TB medicines, improve treatment outcomes, and prevent drug resistance. They cover the following key activities:

1) Integration of TB into national AMR action plans.
2) Implementation of TB surveillance systems.
3) Education of health care providers, individuals affected by TB, and the public.
4) Integration of TB into AMS activities.
5) Establishment of expert consultation services.
6) Targeted testing and preventive treatment for individuals at risk for TB.
7) Access to timely and comprehensive drug susceptibility testing (DST).
8) Prioritisation of efficacy, safety, and resistance prevention in TB treatment regimens.
9) Clinical and microbiological monitoring of treatment response.
10) Assessment of adherence, drug exposure, and resistance in treatment failure.

In addition, the document highlights priorities for future research on TB-specific AMS.

## METHODS

A total of 78 international experts in TB and AMS were invited to participate in a structured consensus process.^6,8^ Experts were nominated by the core coordination team (composed of T.T. Brehm, O.W. Akkerman, G. Sotgiu, S. Tiberi, K.-C. Chang, K. Dheda, R. Duarte, D. Vambe, Z.F. Udwadia, D. Chesov, M. Mendelson, C. Pulcini, and C. Lange) to ensure broad representation across geographic regions, TB incidence levels, country income groups, and professional disciplines. Of the 78 invitees, 9 declined to participate and 7 did not respond after two reminders. The remaining 62 experts were asked to evaluate an initial draft of 10 proposed clinical standards, developed by the core coordination team through informal expert consultations. The panel included representatives from all six WHO regions and 32 countries, 11 of which are classified by the WHO as high-burden countries for TB, TB/HIV, or MDR/RR-TB. Each standard was rated using a 5-point Likert scale (5 = strong agreement; 1 = strong disagreement). According to the predefined consensus threshold, a standard was considered adopted if ≥90% of experts rated it 3 or higher. A draft manuscript was prepared by the core coordination team. The final version was circulated to all participating experts and informally endorsed without further formal voting.

## RESULTS

In the first Delphi round, all 10 standards exceeded this threshold, with agreement rates over 93% and median ratings of 4 or 5, thereby eliminating the need for a second round (Table 1 and Figure). In a subgroup analysis based on the TB incidence in the experts’ country of clinical practice (low, intermediate, or high), all groups showed high levels of agreement, with a median rating of 4 or 5 and agreement exceeding 91% for all standards (Supplementary Data Tables S1–S3).

**Table 1.** Median Likert ratings, interquartile ranges (IQR), and agreement (score ≥ 3) for each item evaluated in the Delphi process.

Clinical standard	Median rating (IQR)	Agreement score, % (n)
1	5 (4–5)	96.8% (60)
2	5 (5–5)	100% (62)
3	5 (4–5)	96.8% (60)
4	5 (4–5)	96.8% (60)
5	4 (4–5)	93.5% (58)
6	5 (4–5)	96.8% (60)
7	5 (4–5)	100% (62)
8	5 (5–5)	100% (62)
9	4.5 (4–5)	96.8% (60)
10	5 (5–5)	100% (62)

**Figure.**
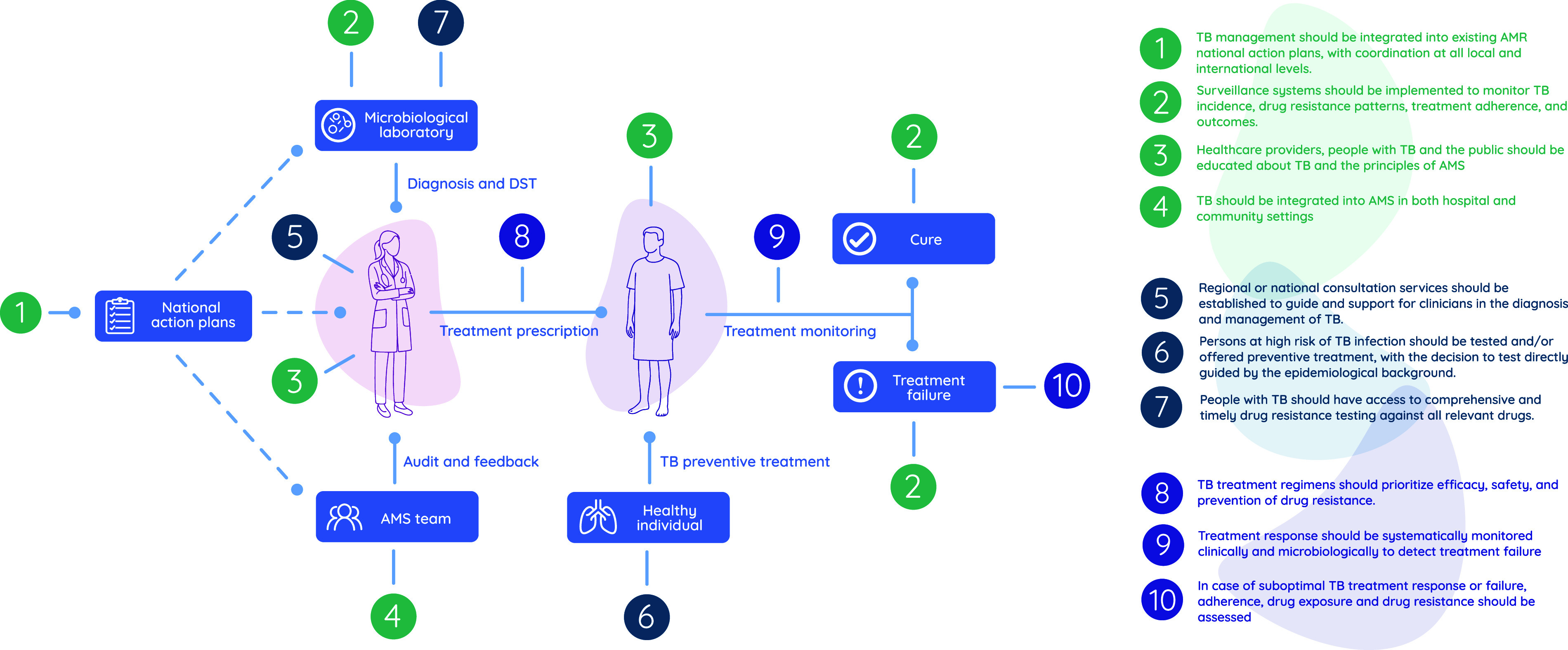
Graphical representation of the 10 clinical standards for antimicrobial stewardship in TB care.

## STANDARD 1

**TB management should be integrated into existing AMR national action plans, with coordination at all local and international levels.**

All countries are encouraged to develop and implement national action plans on AMR that align with the soon-to-be-updated 2015 WHO Global Action Plan on AMR and prioritise interventions likely to have the greatest impact in their specific national context.^9^ In parallel, the WHO launched the End TB Strategy in 2014, which provides the global framework for TB prevention and care.^10^ National action plans must be implemented at both national and local levels. They should comprehensively address infection prevention and care as well as AMS, covering all infections including TB. These plans should also incorporate national policies on active case finding and vaccination to support early detection and prevention of TB. The WHO Global Action Plan ‘covers antibiotic resistance in most detail but also refers, where appropriate, to existing action plans for viral, parasitic and bacterial diseases, including HIV/AIDS, malaria and TB’.^9^ To ensure effective implementation and sustainability, AMR and TB strategies should be aligned and coordinated across international, regional, national, and local levels. However, this alignment remains largely insufficient.

For instance, although the WHO Global Antimicrobial Resistance and Use Surveillance System (GLASS) encourages countries to report data on anti-TB drug use, such reporting is rare. Moreover, the GLASS dashboard currently does not include microbiological TB data.^11^ Similarly, the WHO AMR Country Self-Assessment Survey (TrACSS), which monitors national progress in implementing AMR action plans, does not contain a dedicated item on TB.^12^ While disease-specific policies for TB remain essential, they should not be developed in isolation from broader AMR strategies. Each country must determine the most appropriate model for integration. For example, TB may continue as a distinct programme or pillar, while being explicitly incorporated into the national AMR action plan.

## STANDARD 2

**Surveillance systems should be implemented to monitor TB incidence, *M. tuberculosis* drug-resistance patterns, treatment adherence, and outcomes.**

A robust national TB surveillance system should collect comprehensive epidemiological data from regional and reference TB centres. Timely data collection and analysis are essential to detect changes in incidence – including localised outbreaks or epidemics – identify populations at increased risk of TB infection (TBI) or disease, and monitor the effectiveness of clinical and public health interventions, as well as TB prevention and care programmes. To maximise efficiency and resource utilisation, TB surveillance systems should ideally be integrated with existing public health surveillance platforms, such as those for HIV/AIDS, malaria, diabetes mellitus, and AMR. Such integration would facilitate a coordinated response and optimise the use of human, financial, and logistical resources. In particular, alignment with AMR surveillance is critical to monitoring spatiotemporal trends in drug resistance, thereby informing empiric treatment strategies for both drug-susceptible TB (DS-TB) and DR-TB.

To enhance clinical management of both TB disease and infection, surveillance systems should incorporate variables related to treatment adherence and clinical outcomes. There is a need for international consensus on a core set of indicators to be included in national surveillance frameworks. These should encompass:

I. Notification rates of DS-TB and DR-TB;
II. Adherence metrics, for example, electronic monitoring or prescription record analysis;
III. Demographic data, for example, age, gender, and geographic location;
IV. Clinical characteristics, including symptoms, conventional and molecular bacteriology, risk factors, and genotypic and phenotypic DST results;
V. WHO-defined treatment outcomes.^13^

Furthermore, mechanisms must be established to address common challenges such as under-reporting (e.g., from the private sector), incomplete data, and reporting delays.

## STANDARD 3

**Health care providers, people with TB, and the public should be educated about TB and the principles of AMS.**

The improper use of anti-TB drugs is the primary driver of DR-TB. Education must begin with physicians and other health care providers. A core principle is to prescribe anti-TB drugs only when clearly indicated, using the correct combination, dosage, and duration – ideally as a fixed-dose combination when available. Despite existing guidelines, misprescription remains common, particularly in DR-TB cases, where regimens are more complex and DST for fluoroquinolones and other second-line agents is often not readily accessible. This contributes to the development of additional resistance.^14^ The problem is further exacerbated in the private sector, where some physicians may lack adequate training in TB management.^15^ Policymakers and health care workers must work together to ensure the responsible prescribing and use of anti-TB drugs. Accreditation, education, and awareness programmes should be established to train health care workers across both public and private sectors. Legislation must ensure that only qualified and educated practitioners are allowed to dispense anti-TB medications. This highlights the need for collaborative research and coordinated action between the TB and AMR communities to address the growing challenge of resistance. Education at the patient level is equally important. Adherence to TB treatment is often difficult due to the long treatment duration and potential side effects. Tailored health education and counselling, adapted to the patient’s level of health literacy, is a critical component of TB care. It also provides an opportunity to identify and address individual challenges encountered before and during treatment.^16^ Public education is also essential. In high-risk settings, TB-specific education should be sustainable and behaviour-focused, and use non-stigmatising language in order to effectively promote TB treatment adherence.^17,18^

## STANDARD 4

**TB should be integrated into AMS activities in both hospital and community settings.**

AMS programmes for bacterial infections outside of TB have been shown to reduce AMR and improve patient safety.^19^ Integrating TB-specific AMS into broader AMS initiatives is essential to improving TB treatment outcomes and limiting the emergence of resistance. The availability of a human workforce to implement AMS programmes depends on resource allocation and the setting – hospital versus community. Ideally, TB-specific AMS teams should include infectious disease specialists, pulmonologists, microbiologists, pharmacists, and nurses. In hospital settings, infection prevention and control professionals should also be integral team members.^20,21^ TB-specific AMS relies on accurate diagnosis to determine whether treatment is indicated. If TB is not confirmed, therapy should be withheld whenever possible while alternative diagnoses are explored. If confirmed, DST should be conducted to guide regimen selection for DS- or DR-TB. Another key component is the avoidance of unnecessary antibiotics in patients with unrecognised TB, who are often mistakenly treated for other infections. TB should be considered early in the differential diagnosis, and appropriate tests ordered promptly to avoid diagnostic delays and missed opportunities for effective care. Once TB has been confirmed, the dosage, frequency, duration, and route of administration must be optimised according to the specific regimen and clinical scenario. Integrating TB-specific AMS with infection prevention and control measures is essential to optimise treatment outcomes and prevent transmission across all care settings.^19,22^

## STANDARD 5

**Regional or national consultation services should be established to guide and support clinicians in the diagnosis and management of TB.**

National and regional consultation services, such as expert treatment boards and consilium services, are vital components of TB prevention and care programmes worldwide. They must be designed to be both adaptable and scalable, addressing the specific needs of diverse health care contexts.^23–26^ Their primary goals are to ensure timely and accurate diagnosis, appropriate therapy, and continuous monitoring, thereby reducing TB-related morbidity and mortality and limiting transmission. Evidence from both high- and low-resource settings consistently shows that multidisciplinary stewardship programmes – bringing together TB clinicians, pharmacists, laboratory specialists, nursing staff, field workers, and representatives of affected populations – result in more appropriate and targeted TB treatment.^27^ This leads to improved treatment outcomes, while reducing drug-related toxicity and preventing the development of resistance. The design of TB consultation services should be tailored to the local health care system, available resources, and regional TB incidence. In high-burden countries, referral networks are often established to direct patients to specialised TB centres for expert care. In some settings, telemedicine has been adopted to improve access to expert consultations in remote or underserved areas.^28^ In addition, integrated care models – particularly those that combine TB treatment with services for co-infections such as HIV – provide more comprehensive and effective management.^29^

## STANDARD 6

**Persons at high risk of TBI should be tested and/or offered preventive treatment, with the decision to test directly guided by the epidemiological background.**

Individuals with TBI constitute a reservoir for potential future TB disease and for the development of drug resistance. Therefore, identifying and treating TBI in high-risk groups is a key strategy to reduce TB incidence and progress toward elimination targets.^30–33^ TB is increasingly understood not as a binary state of infection versus disease but as a dynamic spectrum of *M. tuberculosis* activity – ranging from true latency to asymptomatic and symptomatic disease.^34^ This evolving concept reinforces the importance of early identification and targeted preventive strategies, particularly among those at highest risk of progression.^35,36^ Modelling studies further support the population-level impact of targeted testing in reducing TB transmission.^30^ TB preventive treatment in those with TBI should be systematically offered to people living with HIV, close contact persons of individuals with pulmonary TB, those undergoing immunosuppressive therapy (e.g., anti-TNF agents), patients receiving dialysis, candidates for organ transplantation, individuals with silicosis, and migrants from high-burden countries recently arrived in low-burden countries.^31^ However, testing for TBI is not always required before initiating preventive treatment. In groups at particularly high risk of disease progression, such as young child contact persons (<5 years), testing is not routinely recommended prior to treatment initiation, since the risk–benefit ratio clearly favours treatment without prior testing, and testing requirements may delay or prevent access to care. More broadly, in high-incidence settings, testing may be replaced by a broader strategy that offers treatment to all contact persons and high-risk groups.^37^ Importantly, TB preventive treatment should only be initiated after active TB disease has been conclusively ruled out. In areas with a high prevalence of DR-TB, the optimal preventive regimen remains uncertain. Effective implementation of TBI screening requires integration into existing health care platforms – such as HIV, dialysis, or transplant clinics – to ensure seamless linkage to care and follow-up.^36,38^ Equity in access must be a guiding principle, as underserved populations often carry the highest risk and face the greatest barriers to preventive care.^31^

## STANDARD 7

**People with TB should have access to comprehensive and timely drug-resistance testing against all relevant drugs.**

Effective treatment requires rapid and accurate detection of *M. tuberculosis* drug resistance to enable timely and appropriate therapy adjustments based on DST as standard practice (Table 2). Timely DST is critical not only to improve treatment outcomes but also to reduce the transmission of drug-resistant *M. tuberculosis*. The gold standard remains phenotypic DST in liquid or solid culture media, which relies on globally agreed breakpoints to determine drug susceptibility or resistance.^39,40^ However, molecular prediction of resistance offers a faster alternative for guiding treatment decisions.^41^ In most settings, WHO-endorsed near-point-of-care tests can provide adequate prediction for resistance to rifampicin, isoniazid, and fluoroquinolones. However, none of these rapid molecular tests allow prediction of resistance to bedaquiline, pretomanid, or linezolid – key drugs in the WHO-recommended BPaL(M) regimen for the treatment of DR-TB. Additionally, in certain regions, rifampicin-resistance detection via these methods may be unreliable.^42^

**Table 2.** Suggested standard availability of *Mycobacterium tuberculosis* drug-resistance testing.

Type of DST	Time to available test result to the managing physician
Limited near-point-of-care-gDST (Xpert®, LPA) on native material (e.g., sputum and BAL)	2 days
Comprehensive NGS-based gDST on native material (e.g., sputum and BAL)	7 days
Comprehensive pDST on liquid or solid culture, once the culture result is available	14 days

Turnaround times are approximate and refer to the period once relevant material is available. For tNGS, results can usually be reported within 2–4 days once DNA is available, up to 7 days if DNA extraction is required. For WGS, reporting typically requires ≥11–14 days including culture time. For pDST, the culture time has not been included, as it varies considerably depending on the clinical and laboratory context.

BAL = broncho-alveolar lavage; gDST = genotypic drug-susceptibility testing; LPA = line probe assay; pDST = phenotypic drug-susceptibility testing; tNGS = targeted next-generation sequencing; WGS = whole genome sequencing.

Next-generation sequencing (NGS), based on either whole genome sequencing (WGS) or targeted (amplicon-based) NGS (tNGS), offers a more comprehensive and accurate prediction of drug resistance.^41^ While WGS is typically performed on cultured isolates, tNGS can be applied directly to primary clinical specimens. tNGS is currently the most promising technique for the rapid detection of resistance to new and repurposed drugs, and was recommended by the WHO in 2023.^43^ Several tNGS-based assays are already available or in development and may substantially reduce turnaround times compared to culture-based methods. The WHO has developed a standardised catalogue of resistance-associated mutations in the *M. tuberculosis* genome to support consistent interpretation of molecular DST results.^44^ WGS, when interpreted using this catalogue, can provide additional information beyond drug resistance – including strain lineage and transmission dynamics. However, due to its higher cost and requirements for laboratory infrastructure, culture facilities, and specialised training, WGS is currently more feasible in high-resource settings. Given its potential for detailed resistance profiling, cost-effective implementation of molecular DST through NGS should be prioritised to strengthen TB prevention and care efforts.^45^ Importantly, diagnostic tests must be affordable, as they are often paid for out of pocket in low- and middle-income countries – posing a significant barrier to access.

## STANDARD 8

**TB treatment regimens should prioritise efficacy, safety, and prevention of drug resistance.**

TB treatment regimens should demonstrate proven efficacy, characterised by strong bactericidal activity and sufficient sterilising potential to ensure the effective eradication of *M. tuberculosis*.^46^ This requires the use of drugs supported by evidence from clinical trials and real-world treatment programmes, and which are recommended by international treatment guidelines.^47^ Patient safety is equally critical. Regimens should minimise adverse events to support adherence and safeguard patient well-being. While some highly effective drugs – such as linezolid – are associated with a greater risk of toxicity, the balance between efficacy and safety must be carefully weighed. In cases where adverse effects are likely, close monitoring and timely supportive care should be implemented.^48^

Preventing the development of DR-TB depends on both ensuring adherence and using drugs with a high barrier to resistance. For example, while bedaquiline is highly effective, resistance can develop rapidly if adherence is poor. Its use should therefore always be in combination with other active drugs, guided by molecular or phenotypic DST or WGS, to optimise regimen design and reduce the risk of resistance amplification.^49^ Effective treatment must be financially accessible, particularly in settings where out-of-pocket expenditure is common and may lead to poor adherence or treatment interruption. Catastrophic health costs remain a major barrier to treatment completion in many high-burden countries.^50^ Moreover, logistical challenges – including stockouts of essential medications – continue to undermine the reliable delivery of high-quality TB care.^51^

By addressing efficacy, safety, resistance prevention, affordability, and availability, TB treatment regimens can be developed that are not only clinically sound but also sustainable and equitable. However, it must be acknowledged that not all regimens perform equally well across these dimensions, and trade-offs between efficacy, safety, and resistance prevention are sometimes unavoidable in clinical decision making.

## STANDARD 9

**Treatment response should be systematically monitored clinically and microbiologically to detect treatment failure.**

Comprehensive, structured monitoring forms the backbone of high-quality TB care. It ensures complete recovery, minimises relapse, and prevents the emergence and transmission of drug-resistant *M. tuberculosis* strains.^52^ Minimising resistance amplification during treatment requires avoiding subtherapeutic drug concentrations at the site of disease and ensuring early detection of treatment failure to prevent onward transmission of resistant strains.^53^ Monitoring begins with clinical evaluation at every visit, including assessment of symptoms and screening for adverse drug reactions – ideally using a standardised checklist (Table 3).^54^ Bacteriological monitoring, through sputum smear and culture, should be repeated after the intensive phase and at treatment completion. In MDR/RR-TB, sputum culture should be performed in addition to smear microscopy to evaluate treatment response, with monthly cultures being desirable where feasible.^55^ Radiological monitoring with chest X-ray should ideally be conducted at diagnosis, at 2 months, and at the end of treatment. Culture and DST remain essential to assess bacterial viability and detect emerging resistance. Conventional molecular tests, which detect *M. tuberculosis* DNA, are not suitable for monitoring treatment response, as DNA from non-viable bacilli may persist and lead to misinterpretation. However, in real-world settings, limited resources and insufficient political or institutional commitment often constrain the implementation of the suggested comprehensive strategies – despite their clear clinical benefits. In the future, non-sputum-based and culture-free methods (e.g., urine- or blood-based biomarkers and host-response assays) may provide promising alternatives for treatment monitoring, particularly in patient groups where sputum collection is not feasible.^56^

**Table 3.** Suggested standards for treatment monitoring in TB as part of antimicrobial stewardship.

Monitoring aspect	Method	Frequency
Symptoms/adherence	Clinical visit	Monthly or as per schedule
Bacteriology	Sputum smear and culture	Baseline, 2 months, end of treatment
Radiology	Chest radiography	Baseline, 2 months, 6 months, end of treatment if available
Drug toxicity	Blood tests (e.g., liver function and renal function)	Baseline and as indicated
HIV status	Rapid test	Baseline, with ongoing HIV care if positive

## STANDARD 10

**In case of suboptimal TB treatment response or failure, adherence, drug exposure, and drug resistance should be assessed.**

Since 2021, the WHO has updated the definition of treatment failure in TB.^57^ Previously, treatment failure in DS-TB was defined as persistent sputum smear or culture positivity at month 5 or later. The revised definition now includes the need to terminate or permanently modify the treatment regimen or strategy due to a lack of clinical or bacteriological, adverse drug reactions, or the development of acquired drug resistance. Suboptimal treatment response may still be assessed using the pre-2021 definition (i.e., culture positivity at month 5), even if the full criteria for treatment failure are not met. Monitoring treatment response requires a comprehensive evaluation of clinical symptoms, weight changes, chest radiography, and sputum culture.^58^ Treatment failure may also result from selection of novel drug-resistance mutations due to poor adherence during treatment. Also, misleading rifampicin susceptibility results, especially in cases involving resistance-conferring mutations outside the 81-bp hotspot region of the *rpoB* gene, which may not be detected by standard commercial molecular tests or low-level rifampicin resistance, lead to discordant results between molecular and phenotypic DST.^59^ Adherence to treatment is critical, with adherence rates below 90% being associated with worse treatment outcomes, and ensuring treatment adherence remains a cornerstone of TB management.^60^ While directly observed therapy has traditionally been the standard, modern approaches increasingly incorporate patient-centred and community-based support, including psychosocial and material support, and using adherence support tools such as community-based follow-up, in-person supervision, or digital adherence technologies such as video-observed therapy or mobile phone reminders.^55,61,62^ In addition, therapeutic drug monitoring (TDM) should be considered – where available – in cases of suboptimal treatment response or suspected treatment failure, particularly to assess for malabsorption or subtherapeutic drug exposure.^63,64^

## RESEARCH PRIORITIES

Future research should focus on evaluating models for implementing TB-specific AMS across diverse health system contexts, including low- and middle-income countries with high TB burden. Priorities include assessing the effectiveness and cost-effectiveness of AMS interventions; developing and validating rapid, comprehensive, and affordable drug susceptibility tests, optimising strategies for treatment monitoring, adherence support, and TDM, and exploring how to best involve communities, patients, and non-clinical stakeholders in the design and delivery of AMS programmes.

## LIMITATIONS

The proposed clinical standards have several limitations. The expert panel was predominantly composed of clinicians, with limited representation from other key stakeholders such as pharmacists, microbiology laboratories, public health authorities, and people affected by TB. Participants were also skewed toward Europe and low-incidence countries, with underrepresentation from high-burden regions, which may limit applicability in low- and middle-income settings. Nominations were made by the core coordination team with the aim of covering a wide range of clinical and scientific perspectives; however, we agree that diversity in terms of geography and professional disciplines was not fully achieved. Consensus was reached in the first Delphi round, reducing opportunities for iteration. Finally, implementation will depend heavily on local resources, infrastructure, and health system organisation, and evidence for some recommendations – such as culture-free monitoring or large-scale use of tNGS and digital adherence technologies – remains limited.

## CONCLUSION

These clinical standards were developed through international consensus and offer a practical framework for integrating AMS principles into TB care. Implementation must be adapted to local legal, organisational, and resource conditions. Strengthening AMS in TB care is essential to improve outcomes, prevent resistance, and advance global efforts to end TB.

## Supplementary Material


