# Supplementary material for: Clinical standards for antimicrobial stewardship in TB care
Source: IJTLD Open. 2025 Dec 10;2(12):716–26. doi: 10.5588/ijtldopen.25.0522 (PMC12699960; doi:10.5588/ijtldopen.25.0522)
Supplement: Supplementary file 1 [file ijtldopen25-0522_supplementarydata1.pdf]

Supplementary material

Clinical Standards for Antimicrobial Stewardship in Tuberculosis

Thomas Theo Brehm[1,2,3], Onno W Akkerman[4,5], Giovanni Sotgiu[6,7], Simon Tiberi[8], Kwok-Chiu Chang[9], Keertan Dheda[10,11], Raquel Duarte[12,13,14,15], Debrah Vambe[16,17], Zarir F. Udwadia[18], Dumitru Chesov[1,2,19], Marc Mendelson[20], Antonia Morita Saktiawati[21,22], Jakko van Ingen[23], Fusun Oner Eyuboglu[24,25], Thomas Tängdén[26,] Luan Nguyen Quang Vo[27,28], Niccolò Riccardi[29,30], Charalampos Moschos[31], Jon S Friedland[32], Troels Lillebaek[33,34], Sujith J Chandy[35], José A Caminero[36,37], Guy Thwaites[38], Sumanth Gandra[39,40], Karin Thursky[41,42,43], Ige A George[44], Olha Konstantynovska[45,46,47,48], Razia Fatima[49], Jae-Joon Yim[50,51], Nakwon Kwak[52], Ioana Diana Olaru[53], Stephen Gillespie[54], Yousra Kherabi[55,56], Sivan Haia Perl[57,58,59], Erlend Grønningen[60], Camilla Rodrigues[61], Stephanie Bjerrum[62], Franz Bange[63], Vivian Cox[64], Daniela Maria Cirillo[65], Francesca Saluzzo[65], Gabriel Levy Hara[66], Dirk Wagner[67], Nabila Ismail[68], Derek J Sloan[69,70], Ingrid Eshun-Wilsonova[71], Mei Zeng[72], Chloé Cantero[73,74], Tuula Vasankari[75,76], Anna Mandalakas[1,16], Alexander Kay[16,17], Tara Ness[16,77], Marcela Muñoz Torrico[78], Gunar Günter[79,80], Liga Kuksa[81], Lorenzo Guglielmetti[82,83], Alberto L García-Basteiro[84,85,86], Guy B Marks[87,88], Céline Pulcini[89,90], Christoph Lange[1,2,17,91,92]

[1] Department of Clinical Infectious Diseases, Research Center Borstel, Leibniz Lung Center, Borstel, Germany

[2] German Center for Infection Research (DZIF), Partner Site Hamburg-Lübeck-Borstel-Riems, Germany

[3] Division of Infectious Diseases, I. Department of Internal Medicine, University Medical Center Hamburg-Eppendorf, Hamburg, Germany
[4] Department of Pulmonary Diseases and Tuberculosis, University of Groningen, University Medical Center Groningen, Groningen, The Netherlands
[5] Tuberculosis Center Beatrixoord, University of Groningen, University Medical Center Groningen, Haren, The Netherlands
[6] Clinical Epidemiology and Medical Statistics Unit, Department of Medicine, Surgery and Pharmacy, University of Sassari, Sassari, Italy
[7] StopTB Italia ODV, Milan, Italy
[8] Blizard Institute, Barts and The London School of Medicine and Dentistry, Queen Mary University of London, London, UK
[9] Tuberculosis and Chest Service, Centre for Health Protection, Department of Health, Hong Kong, SAR, China
[10] Centre for Lung Infection and Immunity, Division of Pulmonology, Department of Medicine and UCT Lung Institute & South African MRC/UCT Centre for the Study of Antimicrobial Resistance, University of Cape Town, Cape Town, South Africa
[11] Faculty of Infectious and Tropical Diseases, Department of Immunology and Infection, London School of Hygiene and Tropical Medicine, London, UK
[12] EPIUnit, Instituto de Saúde Pública da Universidade do Porto, Porto, Portugal
[13] Laboratório associado para a Investigação Integrativa e Translacional em Saúde Populacional (ITR) Porto, Portugal
[14] Instituto de Ciencias Biomédicas Abel Salazar - ICBAS - Universidade do Porto, Porto, Portugal
[15] Instituto Nacional de Saúde Doutor Ricardo Jorge - INSA-Porto - Porto, Portugal

[16] The Global Tuberculosis Program, Department of Pediatrics, Baylor College of Medicine, Houston, TX 77030, USA

[17] Baylor Children's Foundation-Eswatini, Mbabane, Eswatini

[18] P.D. Hinduja National Hospital and Medical research Centre, Mumbai, India

[19] Discipline of Pneumology and Allergology, Nicolae Testemitanu State University of Medicine and Pharmacy, Chisinau, Republic of Moldova

[20] Division of Infectious Diseases and HIV Medicine, Department of Medicine, University of Cape Town, Cape Town, Western Cape, 7925, South Africa

[21] Department of Internal Medicine, Faculty of Medicine, Public Health, and Nursing, Universitas Gadjah Mada, Yogyakarta, Indonesia.

[22] Center for Tropical Medicine, Faculty of Medicine, Public Health, and Nursing, Universitas Gadjah Mada, Yogyakarta, Indonesia.

[23] Department of Medical Microbiology, Radboud University Medical Center, Nijmegen, the Netherlands

[24] FOE Respiratory Clinic, Ankara, Turkey

[25] Baskent University,Division of Pulmonary Diseases, Ankara, Turkey

[26] Department of Medical Sciences, Uppsala University, Uppsala, Sweden

[27] Friends for International TB Relief, Ha Noi, Viet Nam

[28] Karolinska Institutet, Department of Global Public Health, Stockholm, Sweden

[29] TB Reference Center and Laboratory, ASST Grande Ospedale Metropolitano Niguarda, Milan, Italy

[30] StopTB Italia ODV, Milan, Italy

[31] "Sotiria" Hospital for Chest Diseases, Anti-Tuberculosis Department and Drug Resistant TB Unit

[32] Institute of Infection & Immunity, City St. George's, University of London, London, UK
[33] International Reference Laboratory of Mycobacteriology, Statens Serum Institut, Copenhagen, Denmark
[34] Global Health Section, Department of Public Health, University of Copenhagen, Copenhagen, Denmark.
[35] Department of Pharmacology & Clinical Pharmacology, Christian Medical College, Vellore, India
[36] Pneumology Department, Universitary Hospital of Gran Canaria "Dr. Negrín", Las Palmas GC, Spain
[37] Director of Scientific Activities, ALOSA TB ACADEMY
[38] Oxford University Clinical Research Unit, Ho Chi Minh city, Vietnam
[39] Division of Infectious Diseases, Washington University School of Medicine
[40] Associate Hospital Epidemiologist, Barnes-Jewish Hospital, Saint Louis, MO 63110 USA
[41] Director RMH Guidance Group
[42] Director, National Centre for Antimicrobial Stewardship, Department of Infectious Diseases, University of Melbourne
[43] Centre for Health Services Research in Cancer, Peter MacCallum Cancer Centre
[44] Division of Infectious Disease, Washington University School of Medicine , St Louis, MO, USA
[45] V. N. Karazin Kharkiv National University, Department of Infectious Diseases and Clinical Immunology, Kharkiv, Ukraine
[46] Regional Phthisiopulmonological Center, Kharkiv, Ukraine
[47] Imperial College London, Department of Infectious Diseases

[48] LLC "FH Clinic", Kharkiv, Ukraine

[49] Technical advisor consultant TB strategic planning and Global Fund applications UNOPS

[50] Department of Internal Medicine, Seoul National University College of Medicine, Republic of Korea

[51] Division of Pulmonary and Critical Medicine, Seoul National University Hospital, Republic of Korea

[52] Division of pulmonary and critical care medicine, Department of Internal Medicine, Seoul National University Hospital, Seoul National University College of Medicine, Seoul, South Korea

[53] Clinical Research Department, London School of Hygiene & Tropical Medicine, Keppel Street, London, WC1E 7HT

[54] School of Medicine, North Haugh, St Andrews, Fife, KY16 9T

[55] Infectious and Tropical Diseases Department, Bichat-Claude Bernard Hospital, Assistance Publique-Hôpitaux de Paris, Université Paris Cité, Paris, France

[56] Université Paris Cité, Inserm, IAME, Paris, France

[57] Tuberculosis Departement, Ministry of Health, Israel

[58] Pulmonary Institute, Shamir Medical Center, Zerifin, Israel

[59] Tuberculosis clinic Rehovot, Maccabi Health Services, Israel

[60] Centre for International Health, Department of Global Public Health and Primary Care, Faculty of Medicine, University of Bergen, Norway

[61] Dept Lab Medicine, Hinduja Hospital, Mahim, Mumbai, India

[62] Department of Infectious Diseases, Copenhagen University Hospital, Rigshospitalet, Denmark

[63] Institute for Medical Microbiology and Hospital Epidemiology, Hannover Medical School Hannover, Germany

[64] Johnson & Johnson, Titusville, New Jersey, USA

[65] IRCCS San Raffaele Scientific Institute, Milan, Italy

[66] Unidad de Infectología, Hospital Carlos G Durand, Buenos Aires, Argentina

[67] Division of Infectious Diseases, Department of Internal Medicine II, Freiburg University Medical Centre, Freiburg, Germany.

[68] South African Medical Research Council Centre for Tuberculosis Research, Division of Molecular Biology and Human Genetics, Faculty of Medicine and Health Sciences, Stellenbosch University, Cape Town, South Africa.

[69] School of Medicine, University of St Andrews, Scotland, UK

[70] Dept. of Pulmonology, Radboud University Medical Center, Nijmegen, The Netherlands.

[71] Johnson & Johnson, Cape Town, South Africa

[72] Department of Infectious Diseases, Children's Hospital of Fudan University, National Children's Medical Center, Shanghai 201102, China

[73] Service de Pneumologie, Département de Médecine, Hôpitaux Universitaires de Genève, Genève, Switzerland.

[74] Faculté de Médecine, Université de Genève, Genève, Switzerland.

[75] Department of Pulmonary Diseases and Clinical Allergology, University of Turku, Turku, Finland

[76] Finnish Lung Health Association (FILHA), Helsinki, Finland

[77] Department of Biological Sciences, University of Alaska Anchorage, Anchorage, Alaska, United States of America.

[78] Clínica de Tuberculosis, Instituto Nacional de Enfermedades Respiratorias Ismael Cosío Villegas, Mexico City, Mexico.

[79] Department of Pulmonology, Allergology and Clinical Immunology, Inselspital Bern, Bern University Hospital, Bern, Switzerland.

[80] Department of Clinical Sciences, School of Medicine, University of Namibia, Windhoek, Namibia.

[81] Tuberculosis and Lung Disease clinic, Riga East University hospital, Riga, Latvia.

[82] Médecins Sans Frontières.

[83] Sorbonne Université, National Institute of Health and Medical Research (INSERM), U1135, Centre d'Immunologie Et Des Maladies Infectieuses, Paris.

[84] ISGlobal, Hospital Clínic - Universitat de Barcelona, Barcelona, Spain

[85] Centro de Investigação em Saúde de Manhiça, Maputo, Mozambique

[86] Centro de Investigación Biomédica en Red de Enfermedades Infecciosas, Barcelona, Spain.

[87] Woolcock Institute of Medical Research; No. 298 Kim Ma Street, Building 2G, Ba Dinh District, Hanoi, Vietnam.

[88] Burnet Institute; 85 Commercial Road, Melbourne, Victoria, 3004, Australia.

[89] Université de Lorraine, Inserm, INSPIIRE, Nancy, France

[90] Université de Lorraine, CHRU-Nancy, Centre régional en antibiothérapie du Grand Est AntibioEst, Nancy, France

[91] Respiratory Medicine and International Health, University of Lübeck, Germany

[92] Institute for Infection Research and Vaccine Development (IIRVD), University Medical Center Hamburg-Eppendorf, Hamburg, Germany

Corresponding author

Prof. Dr. med. Dr. h.c. Christoph Lange

Research Center Borstel, Leibniz Lung Center

Tel.: + 49 (0) 4537 188 3010

Email: clange@fz-borstel.de

This document contains the following supplemental materials supporting the article:

Table S1. Median Likert ratings, interquartile ranges (IQR), and agreement (score ≥3) for each item evaluated in the Delphi process from experts from low incidence countries (<10/100.000 population)

Table S2. Median Likert ratings, interquartile ranges (IQR), and agreement (score ≥3) for each item evaluated in the Delphi process from experts from intermediate incidence countries (10-100/100.000 population)

Table S3. Median Likert ratings, interquartile ranges (IQR), and agreement (score ≥3) for each item evaluated in the Delphi process from experts from high incidence countries (>100/100.000 population)

Table S1. Median Likert ratings, interquartile ranges (IQR), and agreement (score ≥3) for each item evaluated in the Delphi process from experts from low incidence countries (<10/100.000 population)

Clinical standard	Median rating (IQR)	Agreement score, % (n)
1	5 (4-5)	91.9% (34)
2	5 (5-5)	100% (37)
3	4 (4-5)	94.6% (35)
4	5 (4-5)	97.3% (36)
5	4 (4-5)	91.9% (34)
6	5 (4-5)	97.3% (36)
7	5 (4-5)	100% (37)
8	5 (5-5)	100% (37)
9	4 (4-5)	94.6% (35)
10	5 (5-5)	100% (37)

Table S2. Median Likert ratings, interquartile ranges (IQR), and agreement (score ≥3) for each item evaluated in the Delphi process from experts from intermediate incidence countries (10-100/100.000 population)

**Clinical standard**	**Median rating (IQR)**	**Agreement score, % (n)**
1	5 (5-5)	100% (10)
2	5 (5-5)	100% (10)
3	5 (5-5)	100% (10)
4	5 (4-5)	100% (10)
5	5 (4-5)	100% (10)
6	5 (4-5)	100% (10)
7	5 (4-5)	100% (10)
8	5 (5-5)	100% (10)
9	5 (4-5)	100% (10)
10	5 (5-5)	100% (10)

Table S3. Median Likert ratings, interquartile ranges (IQR), and agreement (score ≥3) for each item evaluated in the Delphi process from experts from high incidence countries (>100/100.000 population)

Clinical standard	Median rating (IQR)	Agreement score, % (n)
1	5 (4-5)	100% (15)
2	5 (5-5)	100% (15)
3	5 (4-5)	100% (15)
4	4 (3-5)	93.3% (14)
5	5 (4-5)	93.3% (14)
6	4 (4-5)	100% (15)
7	5 (4-5)	100% (15)
8	5 (5-5)	100% (15)
9	5 (4-5)	100% (15)
10	5 (5-5)	100% (15)
